# Prevalence, awareness, treatment, and control of hypertension in community-dwelling older adults with chronic kidney disease: the Irish longitudinal study on ageing

**DOI:** 10.1093/ckj/sfae184

**Published:** 2024-06-27

**Authors:** Leonard D Browne, Mohammed Y Alamin, Hamid H Miri, Robert Hall, Meera Tandan, Donal Sexton, Austin G Stack

**Affiliations:** School of Medicine, University of Limerick, Limerick, Ireland; Health Research Institute (HRI), University of Limerick, Limerick, Ireland; School of Medicine, University of Limerick, Limerick, Ireland; School of Medicine, University of Limerick, Limerick, Ireland; Department of Nephrology, University Hospital Limerick, Limerick, Ireland; School of Medicine, University of Limerick, Limerick, Ireland; Trinity Health Kidney Centre, Trinity College Dublin, Dublin, Ireland; Department of Nephrology, St. James’ Hospital, Dublin, Ireland; School of Medicine, University of Limerick, Limerick, Ireland; Health Research Institute (HRI), University of Limerick, Limerick, Ireland; Department of Nephrology, University Hospital Limerick, Limerick, Ireland

**Keywords:** blood pressure, CKD, epidemiology, guidelines, hypertension

## Abstract

**Background:**

Hypertension is highly prevalent in chronic kidney disease (CKD), posing a significant but modifiable risk for adverse clinical outcomes. This study explored the prevalence, awareness, treatment, and control of hypertension in older Irish adults with CKD.

**Methods:**

Data were analysed from participants in Wave 1 of The Irish Longitudinal Study on Ageing (TILDA) who were aged 50 years and older. CKD was defined as eGFR <60 ml/min/1.72 m^2^, hypertension defined as systolic blood pressure (SBP) ≥140 mmHg and/or diastolic blood pressure (DBP) ≥90 mmHg and/or self-reported use of antihypertensive medication. Participant awareness and treatment of hypertension was based on self-report and SBP/DBP <140/90 mmHg. Multivariable logistic regression examined relationships with awareness, treatment, and control of hypertension expressed as adjusted odds ratios.

**Results:**

Prevalence of hypertension was significantly higher in participants with CKD than without (81.9% vs 59.7%, *P *< .001). Among hypertensive individuals, 70.1% (95% CI: 65.8–74.1) were aware, 83.5% (95% CI 80.0–86.6) were on treatment, yet blood pressure control <140/90 mmHg and SBP <120 mmHg were achieved in only 49.3% (CI 44.0–54.7%) and 17.9% (CI 14.4–22.1), respectively. In multivariable analysis, advancing age 1.05 (CI 1.01–1.10), obesity 6.23 (CI 2.51–15.5), diabetes 5.78 (CI 1.55–21.5), and cardiovascular disease 9.89 (CI 3.27–29.9) were associated with higher odds of treatment, while cardiovascular disease 2.35 (CI 1.39–3.99) and combination antihypertensive therapy 1.76 (CI 1.03–3.01) were associated with blood pressure control.

**Conclusion:**

The prevalence of hypertension is substantial in older Irish adults with CKD; however, control is poor. Approximately, one-third of participants were unaware of their hypertensive status and approximately one-fifth were untreated.

KEY LEARNING POINTS

- Prevalence of hypertension among older Irish adults with CKD is high (81.9%).
- Treatment with multiple antihypertensive medications is common in CKD patients, yet a significant proportion are undertreated.
- Despite high awareness and treatment rates (70.1% and 83.5%, respectively), blood pressure control is poor (49.3% achieving <140/90 mmHg, 17.9% achieving systolic blood pressure <120 mmHg).

**What was known?**

- Hypertension prevalence is high in CKD patients, yet specific data for older Irish adults were lacking.
- Previous studies indicated suboptimal hypertension management in CKD, necessitating further investigation.
- Limited awareness, treatment, and control of hypertension in CKD pose significant challenges to patient outcomes.

**This study adds:**

- High prevalence and poor control of hypertension in older Irish adults with CKD.
- Treatment with multiple antihypertensive medications is common in CKD patients, yet a significant proportion remain undertreated.
- Achievement of recommended blood pressure targets is challenging in CKD, particularly in the elderly.

**Potential impact:**

- The findings underscore the need for more robust quality improvement programmes to improve blood pressure control in CKD.
- The inclusion of hypertension in the new national Structured Chronic Disease Management (CDM) Programme in Ireland should serve to improve the quality of CKD care in these patients.

## INTRODUCTION

Hypertension is common in individuals with chronic kidney disease (CKD), and a major contributor to kidney disease progression, major cardiovascular events, and shortened life spans [1, 2]. Cross-sectional studies both in the general population and in adults with CKD have revealed prevalence in excess of 80% with little change over the past two decades despite advances in therapeutic diagnostics and treatments [3–7]. The principal drivers of worsening hypertension in CKD include salt and water retention, progressive activation of the renin–angiotensin aldosterone system, increased sympathetic activity, and endothelial dysfunction [8]. The relationship between CKD and hypertension is bidirectional, meaning that hypertension can contribute to the progression of CKD, while CKD can also exacerbate pre-existing hypertension. Uncontrolled hypertension confers substantial risk of major cardiovascular events and mortality and is considered a key risk factor in the progression of CKD [9, 10]. Therefore, the treatment of hypertension is an essential component in the overall management of patients with CKD [11].

CKD is a major cause of morbidity and mortality among older individuals [1, 2]. Findings from the Global Burden of Disease Study highlight a global prevalence of 9.1% that contributes to >1.2 million deaths annually, and is destined to increase over time [2]. The coexistence of uncontrolled hypertension and CKD amplifies both the risk of cardiovascular disease and accelerates CKD progression. Accordingly, it is one of the most important modifiable risk factors to protect kidney function and prolong patient survival. Yet our efforts to satisfactorily control blood pressure (BP) have fallen short despite the attention of the international community and recommended clinical guidelines [3–7, 12–15].

In Ireland, there is little information on the burden of hypertension in CKD, and factors that might influence this. A recent study from the Irish Longitudinal Study on Aging (TILDA), a nationally representative sample of community-dwelling adults aged 50 and over, reported a prevalence of 67%. However, levels of awareness were markedly reduced at 54.5% and BP control was achieved in <50% [16]. Whether these findings extend to patients with CKD is unclear.

This study sought to estimate the prevalence, awareness, treatment, and control of hypertension in a nationally representative sample of older persons with CKD in Ireland. A secondary objective was to explore relationships of socioeconomic, clinical, and treatment factors with each of these entities to better understand the burden and treatment of hypertension and to inform national policy.

## MATERIALS AND METHODS

### Study Design and Participants

This cross-sectional study used data from the first wave of TILDA. TILDA is a population-based prospective cohort study, representative of the community living older population in Ireland [17, 18]. The sample was recruited based on a national directory of residential addresses using the RANSAM system [19]. Each member of the population in Ireland aged 50 years and older had an equal probability of being invited to participate.

### Data Collection and BP Measurement

Data collection for the first wave took place between October 2009 and July 2011. Participants completed a computer-assisted face-to-face interview in their home, a self-complete postal questionnaire and a centre or home-based health assessment [17, 18]. Participants aged 50 years or more who had completed the face-to-face interview in their home, a self-complete postal questionnaire, and a centre or home-based health assessment were included. Participants who did not have complete BP measurements and serum creatinine measurements were excluded.

BP was measured by a nurse according to a standard protocol at an ambient temperature of 20–25°C. After a period of rest, a digital automated oscillometric BP monitor (Omron M10-IT, Omron Inc., Kyoto, Japan) with an arm cuff (22–42 cm) was used to measure BP in one arm, at heart height, while the respondent was seated comfortably in an upright position. BP was recorded twice while seated with a timed interval of 1 min between readings. The mean systolic BP (SBP) and diastolic BP (DBP) readings were obtained from these two measurements.

### Hypertension

Hypertension prevalence was defined as SBP ≥140 mmHg or DBP ≥90 mmHg and/or currently taking antihypertensive medications. Awareness of hypertension was defined from self-report; respondents were asked if a doctor ever told them they had ‘high blood pressure or hypertension’. Current antihypertensive medication use was recorded during the home-based interview and classified according to Anatomical Therapeutic Chemical (ATC) classification system. Treatment of hypertension was ascertained using the following antihypertensive medication ATC codes: C02, C03, C07, C08, and C09. BP control was defined as SBP <140 mmHg and DBP <90 mmHg while on antihypertensive medication. An additional threshold target of SBP <120 mmHg was also applied as recommended by the Kidney Disease Improving Global Outcomes (KDIGO) 2021, given that lower SBP <120 mmHg has been found to reduce cardiovascular morbidity and all-cause mortality [12].

Estimated glomerular filtration rate (eGFR) was calculated from serum creatinine using the CKD-EPI equation [20]. CKD was defined as eGFR <60 ml/min/1.72 m^2^. For measurement of filtration markers, a 25 ml venous blood sample was collected from each participant who provided written informed consent during the Wave 1 health assessment. Blood samples were taken during the home assessment as well as the centre-based assessment. All samples were transported to a central laboratory in temperature-controlled shipping boxes for processing within 48 hours. Each sample was centrifuged and aliquoted into 10 bar-coded cryovials that were then stored at −80°C. Cystatin-C and creatinine were measured simultaneously from frozen plasma. Cystatin-C was measured using a second-generation particle enhanced immunoturbidimetric assay (Roche Tina-quant™) on a Roche Cobas 701 analyser. Creatinine was measured using an enzymatic method traceable to isotope-dilution mass spectrometry (Roche Creatinine plus v.2, Roche Diagnostics, Basel, Switzerland).

### Covariates

Demographic covariates included age, sex, highest educational attainment (primary, secondary, and tertiary), and geographical location (Dublin city or county, another town/city, or a rural area). Diabetes was defined by self-reported doctors’ diagnosis or taking diabetes medication (ATC A10A or A10B) or HbA1c ≥6.5% (48 mmol/mol). Cardiovascular disease (CVD)(angina, myocardial infarction, coronary artery bypass surgery, angioplasty/stent insertion, stroke, or transient ischaemic attack) was based on reporting ever having a doctor's diagnosis of these conditions or a self-report of having undergone a procedure. Height and body weight were measured by the study nurse. Body mass index (BMI) was calculated, and participants were classified as underweight (BMI <18.5 kg/m^2^), normal weight (BMI ≥18.5 and <25 kg/m^2^), overweight (BMI ≥25 and <30 kg/m^2^), or obese (BMI ≥30 kg/m^2^).

Physical activity was self-assessed using the International Physical Activity Questionnaire short form (20). The International Physical Activity Questionnaire scoring protocol was used to categorize physical activity as low (light-intensity), moderate (moderate-intensity), or high (vigorous-intensity). Access to subsidized healthcare was captured by the presence of a Medical or General Practitioner (GP) visit card that indicates individuals who are entitled to free or subsidized public healthcare. In Ireland, a medical card entitles the holder to a range of health services free of charge. This includes visits to a GP, prescribed drugs and medicines (with some exceptions), certain dental, optical, and aural services, hospital care, and other community health and personal social services. Eligibility is primarily based on income, with exceptions for certain illnesses or disabilities. A GP Visit Card entitles the holder to free GP visits but does not cover prescribed drugs, hospital care or other services. It has a higher income threshold than the medical card, and all individuals aged 70 and over qualify regardless of income [21].

### Statistical analysis

Descriptive statistics were used to calculate the prevalence, awareness, and control of hypertension. Crude prevalence was calculated with survey weights applied to the estimates to adjust for selection bias and additional weights to reduce non-response bias to the health assessment component of the survey. The population weights were calculated based on age, sex, and educational attainment of the population in Ireland. Statistical significance was set at *P *< .05. Multivariable logistic regression explored associations of demographic, clinical, and behavioural characteristics with hypertension awareness, treatment, and control in separate statistical models. Selection of variables for inclusion in the model was based on perceived importance from literature review or if univariate analysis indicated a significant association. Associations were expressed using adjusted odds ratios (OR) with 95% confidence intervals (CIs). Standard errors are computed with consideration of the loss of precision introduced by sampling weights. The statistical software R was used to conduct the analyses using the package ‘srvyr’ to estimate weighted prevalence [22].

## RESULTS

**Figure 1:**
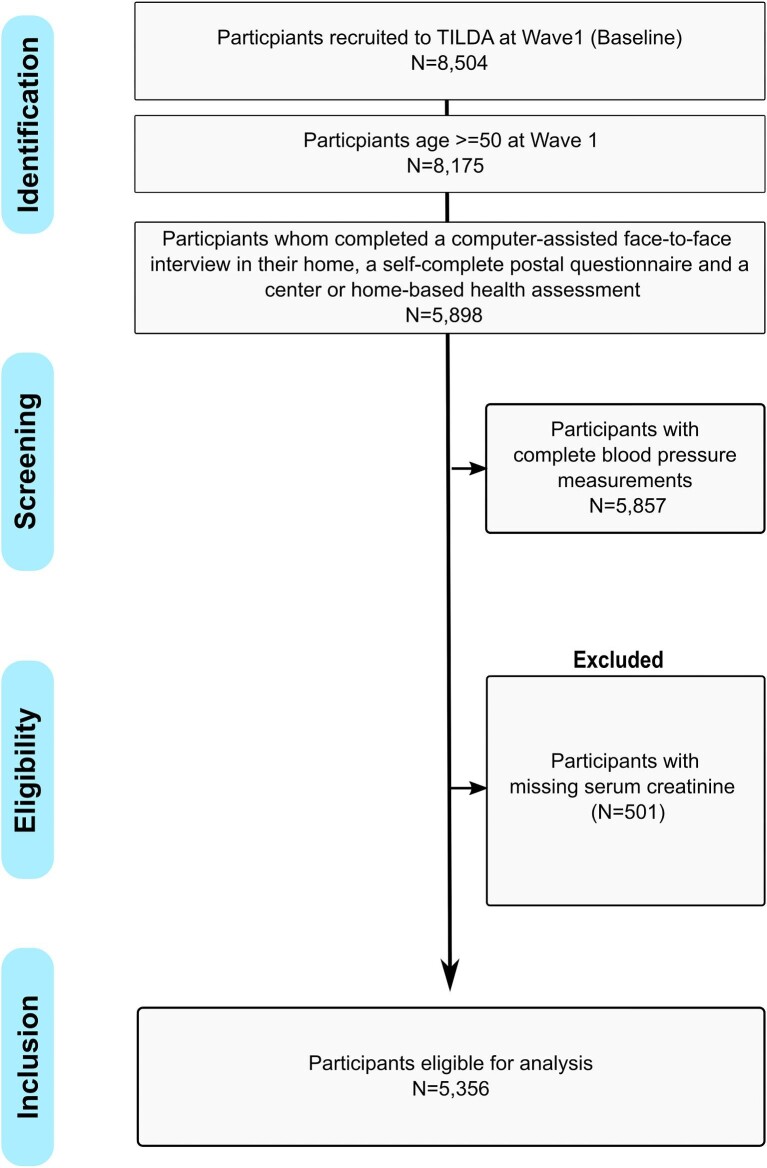
Strobe diagram of study participants from The Irish Longitudinal Study on Ageing (TILDA) cohort aged >50 years who completed a computer-assisted face-to-face interview in their home, a self-complete postal questionnaire and a centre or home-based health assessment and have complete BP and serum creatinine measurements.

In the final analysis, 5356 participants were included after excluding individuals with missing data (Fig. 1). The median age of participants was 62.0 (IQR: 56.0–70.0); 51% were women and 14.0% (95%CI: 12.5–14.6) of the population had CKD. The median eGFR was 51.1 (42.7–56.8) ml/min/1.73 m^2^ in participants with CKD versus 84.5 (74.8–94.3) ml/min/1.73 m^2^ in those without CKD. Compared to individuals without CKD, individuals with CKD were older, had higher comorbidity burden, lower levels of educational attainment, and had higher proportion of individuals with a Medical or GP visit card as shown in Table 1.

**Table 1:** Demographic, health, and behavioural characteristics of the study population with and without CKD.

Characteristic	Overall^a^		Non-CKD^a^		CKD^a^		*P* value^b^
Median age	5356	62.0 (56.0–70.0)	4710	60.0 (55.0–67.0)	646	75.0 (67.0–80.0)	<.001
**Age group**							<.001
50–64	3252	59.9 (58.1–61.7)	3114	66.3 (64.5–68.1)	138	19.1 (16.0–22.2)	
65–74	1419	23.2 (21.9–24.5)	1183	22.3 (20.9–23.6)	236	29.3 (25.5–33.0)	
75+	685	16.9 (15.5–18.3)	413	11.5 (10.3–12.6)	272	51.6 (47.2–56.0)	
**Sex**							<.001
Men	2492	49.0 (47.8–50.1)	2218	50.3 (49.1–51.5)	274	40.3 (36.8–43.8)	
Women	2864	51.0 (49.9–52.2)	2492	49.7 (48.5–50.9)	372	59.7 (56.2–63.2)	
**Education attainment**							<.001
Primary	1362	37.1 (35.3–39.0)	1124	34.5 (32.6–36.3)	238	54.2 (50.1–58.4)	
Secondary	2206	43.8 (42.3–45.4)	1958	45.4 (43.7–47.0)	248	34.1 (30.3–37.9)	
Tertiary	1786	19.0 (17.8–20.2)	1627	20.2 (18.9–21.4)	159	11.6 (9.7–13.5)	
**Location**							.13
Dublin City or County	1396	24.6 (21.0–28.1)	1235	24.8 (21.2–28.4)	161	23.1 (18.2–27.9)	
Another town/city	1480	27.4 (24.1–30.8)	1315	27.8 (24.4–31.2)	165	24.8 (20.1–29.6)	
Rural area	2475	47.9 (44.1–51.7)	2156	47.3 (43.4–51.1)	319	51.9 (46.4–57.5)	
**Health service access**							<.001
Medical or GP visit card	2315	48.6 (46.7–50.4)	1858	44.1 (42.2–46.0)	457	77.2 (73.8–80.5)	
**History of smoking**							<.001
Never	2422	44.0 (42.4–45.5)	2125	43.5 (41.8–45.1)	297	47.1 (42.8–51.5)	
Past	2098	39.2 (37.7–40.7)	1816	38.7 (37.1–40.4)	282	42.2 (38.1–46.2)	
Current	836	16.8 (15.6–18.0)	769	17.8 (16.5–19.1)	67	10.7 (8.2–13.2)	
**BMI kg/m²**							<.001
Underweight	26	0.5 (0.3–0.7)	22	0.5 (0.3–0.7)	4	0.7 (−0.0–1.4)	
Normal	1190	21.0 (19.8–22.2)	1084	21.8 (20.6–23.1)	106	15.7 (12.7–18.6)	
Overweight	2323	43.3 (41.9–44.7)	2070	44.1 (42.6–45.6)	253	38.0 (34.1–41.9)	
Obese	1804	35.0 (33.7–36.3)	1524	33.4 (32.0–34.8)	280	45.2 (41.1–49.4)	
**Physical activity**							<.001
Low	1546	30.3 (28.7–32.0)	1279	28.3 (26.7–29.9)	267	43.5 (39.2–47.7)	
Moderate	1883	34.1 (32.6–35.6)	1672	34.5 (32.9–36.1)	211	31.8 (27.7–35.9)	
High	1881	34.6 (32.7–36.6)	1717	36.3 (34.3–38.3)	164	24.2 (20.5–28.0)	
**BP**							
Median SBP (mmHg)	5356	135.0 (122.0–148.5)	4710	134.5 (122.0–148.0)	646	139.0 (124.0–154.5)	<.001
Median DBP (mmHg)	5356	82.0 (74.5–89.5)	4710	82.5 (75.5–90.0)	646	79.5 (71.5–87.5)	<.001
**Glomerular filtration rate (ml/min/1.72 m^2^)**							
Median eGFR creatinine	5356	81.6 (69.0–92.8)	4710	84.5 (74.8–94.3)	646	51.1 (42.7–56.8)	<.001
Median eGFR cystatin-C	5356	79.2 (65.1–92.5)	4710	82.4 (70.5–94.7)	646	48.5 (37.4–60.7)	<.001
Median eGFR creatinine-cystatin-C	5356	80.9 (68.3–92.1)	4710	83.6 (74.1–94.1)	646	49.5 (40.2–57.7)	<.001
**Comorbid conditions**							
Hypertension	3226	62.7 (61.3–64.1)	2704	59.7 (58.2–61.2)	522	81.9 (78.8–85.0)	<.001
Diabetes	376	7.6 (6.8–8.3)	289	6.5 (5.8–7.3)	87	14.0 (11.1–17.0)	<.001
Cardiovascular disease	559	11.5 (10.5–12.5)	395	9.1 (8.2–10.0)	164	26.7 (22.9–30.5)	<.001
Angina	264	5.5 (4.8–6.2)	175	4.0 (3.4–4.6)	89	15.2 (12.1–18.3)	<.001
Heart Attack	235	4.8 (4.2–5.5)	165	3.9 (3.3–4.5)	70	11.2 (8.6–13.7)	<.001
Congestive heart failure	52	1.1 (0.8–1.4)	34	0.8 (0.6–1.1)	18	2.8 (1.5–4.2)	<.001
Stroke	80	1.7 (1.3–2.0)	58	1.3 (1.0–1.7)	22	3.7 (2.0–5.4)	<.001
Mini stroke or TIA	107	2.1 (1.7–2.5)	73	1.6 (1.3–2.0)	34	5.0 (3.2–6.8)	<.001
High cholesterol	2187	40.4 (38.9–41.9)	1880	39.3 (37.7–40.8)	307	47.5 (43.5–51.4)	<.001
Chronic lung disease	205	4.2 (3.5–4.8)	181	4.1 (3.4–4.7)	24	4.7 (2.8–6.6)	.5
Cancer	328	6.3 (5.6–7.0)	264	5.6 (5.0–6.3)	64	10.7 (7.9–13.6)	<.001

a Median (IQR), *n* (%) adjusted for oversampling and non-response using sample survey weights.

b Wilcoxon rank-sum test for complex survey samples; chi-squared test with Rao and Scott's second-order correction

Abbreviations: TIA, transient ischaemic attack.

eGFR was estimated using the CKD-EPI equation for serum creatinine concentrations.

### Prevalence, awareness, treatment, and control of hypertension

**Figure 2:**
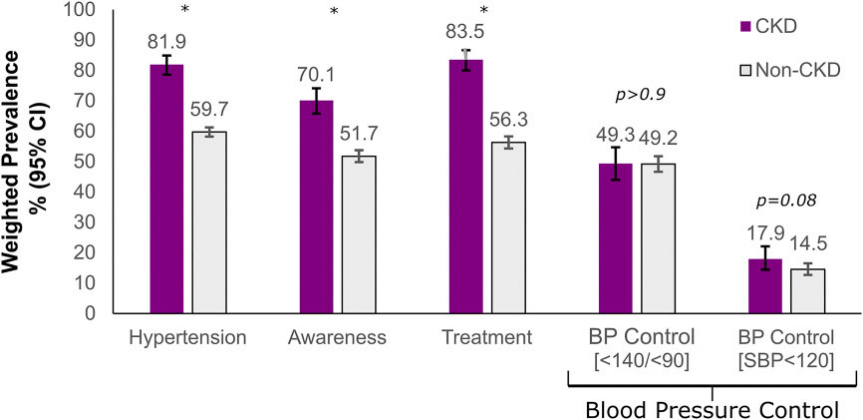
Prevalence of hypertension awareness, treatment, and control by CKD status (*P* value: chi-squared test with Rao and Scott's second-order correction, *indicates *P* < .001).

The weighted prevalence of hypertension was significantly higher in participants with CKD than without CKD (81.9% vs 59.7%, respectively) (Table 1, Fig. 2). Among CKD patients, hypertension was significantly higher in men than in women (88.0% vs 77.9%, *P *= .025). Age specific prevalence was higher for men than women in the 50–64 and 65–74 age groups, but were similar in the oldest age group 75+ (Table 2). Overall, 70.1 (95%CI: 68.7–75.965.8–74.1) of older individuals were aware of their hypertension, and 83.5% (95% CI 80.0–86.6) were treated with antihypertensive medications (Table 3). Hypertension awareness was significantly higher in women than men (74.8% vs 64.0%, *P *= .011) but did not increase significantly with age, varying from 63.6% to 70.3% from the youngest to oldest age groups. Interestingly, the percentage of individuals treated with antihypertensive medications increased in a graded fashion with advancing age (*P *= .004).

**Table 2:** Prevalence of hypertension in older adults with CKD in Ireland by age and sex.

	Women (*N* = 372)		Men (*N* = 274)		All (*N* = 646)	
	% 95% CI	*P* value	% 95% CI	*P* value	% 95% CI	*P* value
**Age group**		<0.001		0.5		<.001
50–64	59.3 (47.7–70.0)		83.8 (70.9–91.7)		69.5 (60.9–76.9)	
65–74	74.2 (65.7–81.1)		89.5 (82.0–94.1)		80.9 (75.2–85.6)	
75+	86.2 (78.8–91.3)		88.6 (81.1–93.4)		87.1 (82.0–90.9)	
**Total**	77.9 (73.0–82.1)		88.0 (83.6–91.3)		81.9 (78.6–84.9)	.025

Hypertension: SBP = 140 mmHg or DBP = 90 mmHg and/or currently taking antihypertensive medications.

Weighted column %, adjusted for oversampling and non-response using sample survey weights.

**Table 3:** Awareness, treatment, and control of hypertension among older adults with CKD in Ireland.

	Awareness % (95% CI)	*P* value	Treatment % (95% CI)	*P* value	BP <140/90 mmHg	*P* value	SBP <120 mmHg	*P* value
	(*N* = 522)		(*N* = 522)		(*N* = 424)		(*N* = 424)	
**Sex**		.011		.7		.6		.9
Men	64.0 (57.2–70.3)		82.9 (77.3–87.3)		50.9 (43.6–58.2)		17.9 (13.0–24.3)	
Women	74.8 (69.2–79.7)		84.0 (79.0–88.1)		48.1 (41.1–55.3)		17.9 (13.3–23.5)	
**Age group**		.4		.004		.006		<.001
50–64	63.6 (52.7–73.3)		71.7 (61.5–80.1)		58.8 (46.4–70.3)		21.4 (12.4–34.2)	
65–74	73.5 (66.7–79.4)		83.9 (77.8–88.6)		58.3 (49.6–66.5)		28.5 (21.9–36.2)	
75+	70.3 (64.0–75.8)		86.8 (81.7–90.7)		42.4 (35.1–50.1)		11.6 (7.8–17.1)	
**Total**	70.1 (65.8–74.1)		83.5 (80.0–86.6)		49.3 (44.0–54.7)		17.9 (14.4–22.1)	

Weighted column %, adjusted for oversampling and non-response using sample survey weights.

Treatment: Indicator of respondents who take any antihypertensive medication with ATC code C02, C03, C07, C08, C09

Respondents were asked if a doctor ever told them they had ‘high blood pressure or hypertension’; awareness of hypertension was defined as endorsement of this item. (ph201_01)

Definition 1: Control of BP was defined as SBP <140 mmHg and DBP <90 mmHg while on antihypertensive medication.

Definition 2: Control of BP was defined as SBP <120 mmHg while on antihypertensive medication.

*P* value: chi-squared test with Rao and Scott's second-order correction.

Despite high levels of awareness and treatment of hypertension in those with CKD, only 49.3% (95% CI 44.0–54.7) of treated subjects achieved BP <140/90 mmHg, with 17.9% (95% CI: 14.4–22.1) achieving SBP <120 mmHg (Table 3). Control rates were poorest for individuals in the 75+ age category compared to younger age groups for either BP <140/90 mmHg (*P* = .006) or SBP <120 mmHg (*P* < .001).

### Treatment with antihypertensive medication in CKD

Among hypertensive individuals with CKD and on antihypertensive treatment, the most frequently prescribed medications were angiotensin-converting enzyme inhibitors or angiotensin receptor blockers (ARB) [66.5% (95% CI: 61.7–71.0%)], followed by β-blockers [42.2% (95% CI: 37.0–47.5%)], diuretics [32.1% (95% CI: 27.3–37.4%)], and calcium channel blockers [28.5% (95% CI: 23.8–33.6%)]. A combination of two or more antihypertensive medications was received by 61.8% (95% CI: 56.8–66.6%) of patients and this proportion increased significantly from 45.9% in the 50–64-year age group to 66.8% in the 75+ age group (Table S1).

### Correlates of hypertension awareness, treatment, and control in CKD.

In multivariable analysis, women were more aware than men [OR, 2.03 (95% CI 1.21–3.41)] as shown in Table 4. Obese individuals [OR 3.25 (95% CI 1.58–6.69)] and those with a history of excess alcohol consumption [OR 3.83 (95% CI 0.38–10.60)] were more aware of hypertension than normal weight individuals or those with low alcohol consumption, and these associations were independent of demographic and other socioeconomic factors.

**Table 4:** Multivariable logistic regression models of awareness, treatment, and control of hypertension in persons with CKD.

	Awareness^a^		Treatment^b^		Control in the treated^d^	
Covariate	OR (95%CI)	*P* value	OR (95%CI)	*P* value	OR (95%CI)	*P* value
Age (years)	1.03 (0.99–1.06)	.2	1.05 (1.01–1.10)	.024	0.94 (0.90–0.97)	.001
**Sex**						
Men (reference)	1.00 (−)		1.00 (−)		1.00 (−)	
Women	2.03 (1.21–3.41)	.008	1.55 (0.76–3.17)	.2	1.45 (0.84–2.50)	.2
**Education**						
Primary (reference)	1.00 (−)		1.00 (−)		1.00 (−)	
Secondary	0.89 (0.54–1.47)	.7	0.83 (0.43–1.61)	.6	1.13 (0.65–1.97)	.7
Tertiary	0.88 (0.46–1.66)	.7	1.01 (0.45–2.27)	>.9	1.23 (0.62–2.44)	.6
**Location**						
Dublin city or county (reference)	1.00 (−)		1.00 (−)		1.00 (−)	
Another town or city	0.64 (0.35–1.18)	.15	1.32 (0.58–3.03)	.5	0.93 (0.45–1.95)	.9
A rural area	0.85 (0.48–1.51)	.6	1.37 (0.70–2.67)	.4	1.25 (0.66–2.37)	.5
**BMI**						
Normal (reference)	1.00 (−)		1.00 (−)		1.00 (−)	
Overweight	1.62 (0.78–3.37)	.2	1.84 (0.84–4.03)	.13	1.37 (0.61–3.08)	.4
Obese	3.25 (1.58–6.69)	.001	6.22 (2.50–15.4)	<.001	1.67 (0.73–3.84)	.2
**Physical activity**						
Low physical activity (reference)	1.00 (−)		1.00 (−)		1.00 (−)	
Moderate physical activity	1.06 (0.60–1.87)	.8	0.70 (0.33–1.49)	.4	1.31 (0.74–2.32)	.4
High physical activity	0.88 (0.50–1.54)	.6	0.40 (0.19–0.86)	.018	1.71 (0.84–3.48)	.14
**Smoking status**						
Never (reference)	1.00 (−)		1.00 (−)		1.00 (−)	
Past	1.32 (0.80–2.18)	.3	1.39 (0.67–2.88)	.4	1.43 (0.81–2.53)	.2
Current	1.19 (0.58–2.44)	.6	1.17 (0.47–2.92)	.7	0.91 (0.34–2.40)	.8
**Alcohol CAGE ≥2**	3.83 (1.38–10.60)	.010	3.22 (0.94–11.10)	.064	0.80 (0.32–2.02)	.6
**Diabetes**	1.92 (0.90–4.11)	.093	5.76 (1.55–21.40)	.009	0.75 (0.37–1.50)	.4
**Cardiovascular disease**	1.35 (0.80–2.28)	.3	9.90 (3.27–30.0)	<.001	2.35 (1.39–3.99)	.002
**Medical or GP visit card**	0.57 (0.29–1.09)	.089	0.43 (0.18–1.01)	.052	1.70 (0.87–3.31)	.12
Combination therapy*					1.76 (1.03–3.01)	.038

a Respondents were asked if a doctor ever told them they had ‘high blood pressure or hypertension’; awareness of hypertension was defined as endorsement of this item (ph201_01).

b Treatment: indicator of respondents who take any antihypertensive medication with ATC code C02, C03, C07, C08, C09.

c BP control was defined as SBP <140 mmHg and DBP <90 mmHg while on antihypertensive medication.

d Combination therapy indicates individuals who were treated with two or more antihypertensive medications, and was included in the logistic model for BP control <140/90 mmHg.

Standard errors are computed with consideration of the loss of precision introduced by sampling weights.

When treatment of hypertension was modelled as an outcome, increasing age [OR 1.05 (95% CI 1.01–1.10)], obesity [OR 6.22 (95% 2.50–15.4)], diabetes [OR 5.76 (95% CI 1.55–21.40)], and cardiovascular disease [OR 9.90 (95% CI 3.27–30.0)] were significantly associated with treatment. Unlike obesity, participants with high levels of physical activity were less likely to be on treatment OR 0.40 (95% CI 0.19–0.86) compared to individuals with lower levels of physical activity.

Finally, when we modelled control of BP (defined as BP values <140/90 mmHg), we found the likelihood of control was lower with advancing age [OR 0.94 (95% CI 0.90–0.97)]. The achievement of BP control was more likely for individuals with a history of cardiovascular disease [OR 2.35 (95% CI 1. 1.39–3.99)] and for those treated with two or more antihypertensive medications [OR 1.76 (95% CI 1.03–3.01)].

## DISCUSSION

This is the first comprehensive study to address the prevalence, treatment, and control of hypertension in older individuals with CKD in Ireland using a nationwide sample of community-dwelling adults. We found that the prevalence of hypertension was far higher in individuals with CKD than without CKD, and this was accompanied with high levels of awareness and treatment. However, despite these positive findings, BP control was far from optimal with 49.3% of patients achieving a target BP of <140/90 mmHg and only 17.9% achieving a SBP <120 mmHg, respectively. Our analysis identified the presence of key demographic, socioeconomic, clinical, and behavioural factors that correlated with awareness of and treatment of hypertension in CKD patients. We confirm that BP control remains a challenging task in CKD especially for older patients even with conservative BP targets.

The primary objective of this study was to better understand the treatment of hypertension in CKD in the Irish population and identify potential determinants across domains of awareness, treatment, and control. Important insights into BP management in the general population of Ireland has previously been reported by Murphy *et al.* [16]. In their analysis of TILDA, awareness rates were 54.5%, treatment rates were 59%, while control of BP to <140/90 mmHg was achieved in 51.6%. In comparison, the present study found much higher levels of awareness and treatment in individuals with CKD than for the general population suggesting better opportunities to improve BP management, however, blood control rates were similarly disappointing (50.4% vs 51.6%). International studies of CKD participants have consistently reported poor rates of BP control [3–6]. Achievement of BP target of <140/90 mmHg in CKD was 48.5%, 45.5%, 49%, and 41% in American, Spanish, German, and Chinese populations, respectively. Taken together, these country-specific studies confirm that guideline-recommended targets for BP control in CKD are not being reached with existing CKD care strategies.

It is noteworthy that only 17.9% of CKD participants in TILDA achieved a target of SBP <120 mmHg, the internationally recommended threshold from the KDIGO Clinical Practice Guideline for the Management of Blood Pressure in Chronic Kidney Disease and the European Society of Cardiology (ESC) 2021 Clinical Guideline on Cardiovascular Disease Prevention in Clinical Practice [12, 23, 24]. The proportion achieving this target was further reduced to 11.6% in the very elderly. The recommendation on a threshold of SBP 120 mmHg was based on subgroup analysis of the SPRINT trial, which revealed significantly lower rates of cardiovascular events and lower overall mortality in patients that were titrated to a lower BP of 120 mmHg versus <140 mmHg after a 3.3-year follow-up. [25]. Notwithstanding these benefits, residual concerns exist among the nephrology community on the generalizability of this guideline along with legitimate concerns of potential harm especially in the elderly from postural hypotension, falls and fractures, acute kidney injury, and stroke [24, 26, 27]. Older adults with CKD often have high BP. While it might seem that aiming for a SBP <120 mmHg could pose risks for these individuals, the SPRINT trial found no significant increase in severe adverse events, such as orthostatic hypotension, syncope, or injurious falls, in the intensive treatment group. However, Sexton *et al.* used TILDA data to assess whether older, community-dwelling Irish adults, who would meet SPRINT inclusion criteria, experienced similar risks outside the clinical trial setting. Their findings indicated that these adults had rates of injurious falls and syncope approximately five times higher than the standard care group in SPRINT, suggesting that intensive hypertension treatment might indeed be harmful in this older population [27]. Moreover, the variability in BP guidelines and the lack of randomized controlled trials investigating intensive BP treatment in older adults add to the complexity of determining optimal treatment targets. A recent review by Verdecchia *et al*. found substantial heterogeneity in the available data supporting fixed BP targets. They suggested that BP targets should be personalized for all patients, reflecting the concept of the lowest well-tolerated BP based on the best trade-off between efficacy and safety [28]. This underscores the importance of individualized treatment plans to balance the benefits and risks of intensive BP management in older adults. Consequently, it is unlikely that the new KDIGO 2021 BP target will be achieved in most patients given these safety concerns and an ageing CKD population.

This study highlights important differences in sociodemographic and clinical characteristics associated with the sequential cascade of hypertension awareness, treatment, and control. Although treatment of hypertension was strongly associated with increasing BMI, physical inactivity, and diabetes, none of these factors was correlated with BP control <140/90 mmHg. Obesity and diabetes are major risk factors for treatment resistant hypertension, which may in part explain the difficulty in achieving BP control [29, 30]. Among TILDA participants with CKD, the proportion of individuals classified as overweight (38%) or obese (45%) was substantial, while 14% had diabetes. We did find that individuals with a history of cardiovascular disease were more likely to receive hypertension treatment and achieve BP control. This observation is consistent with published research and reflects the regular contact of these patients with the health system with greater opportunities to optimize BP management [31].

Hypertension confers the greatest population attributable risk for cardiovascular events than any other modifiable risk factor [32]. Moreover, individuals with CKD are more likely to have resistant hypertension that those without CKD and typically require a combination of antihypertensive medications to ensure effective BP control. Despite the availability of evidence-based clinical guidelines and the acknowledgement that many hypertensive patients require multiple antihypertensive medications to control BP [30], we found that just under two-thirds of individuals with CKD in TILDA were treated with two or more antihypertensive medications suggesting that a large proportion of patients were treated with just a single agent. The proportion on combination therapy was lowest at 45.9% in the 50–64 age group and increased in a graded fashion with advancing age. Furthermore, our multivariable model confirmed that individuals with CKD treated with combination antihypertensive therapy were more likely to achieve BP control taking into consideration demographic and socioeconomic factors. These findings might well indicate possible inertia to treatment escalation or the reluctance to intensify treatment in older individuals due to safety concerns and controversy on BP targets [24, 26, 27, 33].

It is possible that non-adherence to medication at an individual level and the absence of a structured programme for control of BP may contribute to the low levels of control at the population level as observed in this Irish study. In 2017, the Irish Government published Sláintecare, a 10-year programme to deliver health system reform and universal healthcare [34]. As part of this strategy, a Chronic Disease Management Programme (CDM) has been implemented since 2020 for people with specific chronic diseases, and who have a medical card or a GP visit card. This has the potential to further increase access to health care access, and improve the management of hypertension at the population level [35].

Findings from the present analysis must be considered within the context of inherent limitations. This study is observational; therefore, causality cannot be confirmed between explanatory variables and each modelled outcome. We also recognize the limitation of single measurement of creatinine to define CKD presence. The potential for misclassification of hypertension may have occurred in normotensive individuals whose BP tested high during the study, often termed ‘white coat hypertension’ [36]. This study did not explore the indications for which antihypertensive medications were prescribed. In routine clinical practice, medications used in the treatment of hypertension may also have efficacy in the treatment of angina and heart failure and thus have a dual benefit. This lack of detailed information restricts our understanding of the underlying motivations that guided treatment decisions and their correlation with patient characteristics. Finally, we were unable to measure medication adherence, which may in part explain differences in BP control. Nevertheless, the strengths of this study were several. It is representative of the Irish population and the findings are generalizable to community-dwelling adults aged ≥50 years in Ireland. Extensive information was collected on a broad range of demographic, socioeconomic, clinical, behavioural, and lifestyle characteristics through rigorous standardized protocols with very little missing data. These allowed us to explore in depth the relationships with each component of the hypertension cascade pathway from awareness to control.

## CONCLUSION

We found a high prevalence of hypertension in older Irish adults with CKD with suboptimal BP control in over half of participants. Approximately one-third of participants with CKD were unaware of their hypertensive status and approximately one-fifth of these participants were untreated. With an ageing population in Ireland, older people now account for a much greater proportion of patients with or at risk for kidney disease and kidney failure. Improvement of hypertension awareness, treatment, and control should remain a key public health priority to reduce the progression of CKD and associated complications. We advocate the need for more robust quality improvement programmes at the population level to improve BP control. The inclusion of hypertension in the new national Structured CDM Programme in Ireland is a welcome development and should serve to improve the quality of care in these patients.

## Supplementary Material

sfae184_Supplemental_File

## Data Availability

The data underlying this article are available in the article and in its online supplementary material.
