# Supplementary material for: Prevalence, awareness, treatment, and control of hypertension in community-dwelling older adults with chronic kidney disease: the Irish longitudinal study on ageing
Source: Clin Kidney J. 2024 Jun 27;17(8):sfae184. doi: 10.1093/ckj/sfae184 (PMC11292221; doi:10.1093/ckj/sfae184)
Supplement: sfae184_Supplemental_File [file sfae184_supplemental_file.docx]

Table S1. Antihypertensive medication utilization in adults aged 50 years and older with CKD in Ireland (TILDA wave 1, N =522)

|  | **Anti-adrenergic agents** | | **Diuretics** | | **ß-blockers** | | **Calcium channel blockers** | | **ACE inhibitors** | | **ARBs** | | **ACE or ARBS** | | **Combination therapy** | | |
| --- | --- | --- | --- | --- | --- | --- | --- | --- | --- | --- | --- | --- | --- | --- | --- | --- | --- |
|  | **%** | **95% CI** | **%** | **95% CI** | **%** | **95% CI** | **%** | **95% CI** | **%** | **95% CI** | **%** | **95% CI** | **%** | **95% CI** | **%** | **95% CI** |  |
| **Sex** |  |  |  |  |  |  |  |  |  |  |  |  |  |  |  |  |  |
| Male | 8.1 | (4.8-13.3) | 28.9 | (22.6-36.0) | 42 | (34.8-49.5) | 30.5 | (23.9-38.1) | 39 | (32.0-46.4) | 28.5 | (22.3-35.7) | 65.6 | (58.1-72.4) | 60.4 | (52.9-67.5) |  |
| Female | 4.9 | (2.8-8.4) | 34.6 | (28.2-41.7) | 42.3 | (35.4-49.5) | 26.9 | (21.0-33.8) | 30.2 | (23.8-37.5) | 38.3 | (31.6-45.4) | 67.2 | (60.3-73.4) | 62.9 | (56.0-69.3) |  |
| **Age** |  |  |  |  |  |  |  |  |  |  |  |  |  |  |  |  |  |
| 50-64 | 4.2 | (1.3-12.4) | 11.3 | (5.7-21.3) | 38.8 | (27.6-51.3) | 19.1 | (10.9-31.3) | 33.2 | (22.4-46.1) | 33.2 | (23.2-44.8) | 66.3 | (54.1-76.7) | 45.9 | (34.4-57.9) |  |
| 65-74 | 6.1 | (3.2-11.2) | 25.6 | (19.3-33.1) | 39.7 | (31.9-48.0) | 29.4 | (22.1-37.9) | 36.2 | (28.7-44.6) | 38.1 | (30.6-46.2) | 73.4 | (65.5-80.0) | 59.9 | (51.7-67.5) |  |
| 75+ | 6.8 | (4.2-11.0) | 40.6 | (33.2-48.5) | 44.3 | (36.9-51.9) | 30.3 | (23.5-38.1) | 33.1 | (25.9-41.2) | 32.3 | (25.6-39.8) | 63 | (55.8-69.7) | 66.8 | (59.5-73.3) |  |
| Total | 6.2 | (4.3-9.0) | 32.1 | (27.3-37.4) | 42.2 | (37.0-47.5) | 28.5 | (23.8-33.6) | 34 | (28.9-39.5) | 34.1 | (29.3-39.2) | 66.5 | (61.7-71.0) | 61.8 | (56.8-66.6) |  |

Note: ACE=Angiotensin-converting enzyme, ARBs=Angiotensin receptor blockers, Combination therapy included any combination of two or more antihypertensive medications, weighted prevalence calculated with Health assessment weights.
